# A Neutrophil Phenotype Model for Extracorporeal Treatment of Sepsis

**DOI:** 10.1371/journal.pcbi.1004314

**Published:** 2015-10-15

**Authors:** Alexander D. Malkin, Robert P. Sheehan, Shibin Mathew, William J. Federspiel, Heinz Redl, Gilles Clermont

**Affiliations:** 1 McGowan Institute for Regenerative Medicine, University of Pittsburgh, Pittsburgh, Pennsylvania, United States of America; 2 Department of Bioengineering, University of Pittsburgh, Pittsburgh, Pennsylvania, United States of America; 3 Department of Computational and Systems Biology, University of Pittsburgh, Pittsburgh, Pennsylvania, United States of America; 4 Department of Chemical and Petroleum Engineering, University of Pittsburgh, Pittsburgh, Pennsylvania, United States of America; 5 Department of Surgery, University of Pittsburgh, Pittsburgh, Pennsylvania, United States of America; 6 Ludwig Boltzmann Institute for Experimental and Clinical Traumatology in AUVA center, Vienna, Austria; 7 CRISMA Center, Department of Critical Care Medicine, University of Pittsburgh, Pittsburgh, Pennsylvania, United States of America; University of Iowa, UNITED STATES

## Abstract

Neutrophils play a central role in eliminating bacterial pathogens, but may also contribute to end-organ damage in sepsis. Interleukin-8 (IL-8), a key modulator of neutrophil function, signals through neutrophil specific surface receptors CXCR-1 and CXCR-2. In this study a mechanistic computational model was used to evaluate and deploy an extracorporeal sepsis treatment which modulates CXCR-1/2 levels. First, a simplified mechanistic computational model of IL-8 mediated activation of CXCR-1/2 receptors was developed, containing 16 ODEs and 43 parameters. Receptor level dynamics and systemic parameters were coupled with multiple neutrophil phenotypes to generate dynamic populations of activated neutrophils which reduce pathogen load, and/or primed neutrophils which cause adverse tissue damage when misdirected. The mathematical model was calibrated using experimental data from baboons administered a two-hour infusion of E *coli* and followed for a maximum of 28 days. Ensembles of parameters were generated using a Bayesian parallel tempering approach to produce model fits that could recreate experimental outcomes. Stepwise logistic regression identified seven model parameters as key determinants of mortality. Sensitivity analysis showed that parameters controlling the level of killer cell neutrophils affected the overall systemic damage of individuals. To evaluate rescue strategies and provide probabilistic predictions of their impact on mortality, time of onset, duration, and capture efficacy of an extracorporeal device that modulated neutrophil phenotype were explored. Our findings suggest that interventions aiming to modulate phenotypic composition are time sensitive. When introduced between 3–6 hours of infection for a 72 hour duration, the survivor population increased from 31% to 40–80%. Treatment efficacy quickly diminishes if not introduced within 15 hours of infection. Significant harm is possible with treatment durations ranging from 5–24 hours, which may reduce survival to 13%. In severe sepsis, an extracorporeal treatment which modulates CXCR-1/2 levels has therapeutic potential, but also potential for harm. Further development of the computational model will help guide optimal device development and determine which patient populations should be targeted by treatment.

## Author Summary

Sepsis occurs when a patient develops a whole body immune response due to infection. In this condition, white blood cells called neutrophils circulate in an active state, seeking and eliminating invading bacteria. However, when neutrophils are activated, healthy tissue is inadvertently targeted, leading to organ damage and potentially death. Even though sepsis kills millions worldwide, there are still no specific treatments approved in the United States. This may be due to the complexity and diversity of the body’s immune response, which can be managed well using computational modeling. We have developed a computational model to predict how different levels of neutrophil activation impact survival in an overactive inflammatory conditions. The model was utilized to assess the effectiveness of a simulated experimental sepsis treatment which modulates neutrophil populations and activity. This evaluation determined that treatment timing plays a critical role in therapeutic effectiveness. When utilized properly the treatment drastically improves survival, but there is also risk of causing patient harm when introduced at the wrong time. We intend for this computational model to support and guide further development of sepsis treatments and help translate these preliminary results from bench to bedside.

## Introduction

Sepsis, a systemic inflammatory response due to an infection, affects 900,000 Americans per year and its incidence is expected to increase over the next 10–20 years as the population ages [1]. While it is acknowledged that sepsis is a growing problem, its associated mortality rate has remained persistently high for the last 20 years and is currently near 20% [1–4]. Sepsis is now the leading cause of in-hospital death in the United States, yet there are no FDA approved specific treatments [5]. While understanding of the underlying mechanisms in sepsis has been rapidly improving, translation to clinically effective treatments has proven very challenging [6,7]. Much of this difficulty translating treatments may be the diversity and complexity of individual immune response and patient population [8,9]. These complexities lend themselves well to computational modeling, which can help integrate these complexities into a unified pathophysiological framework and optimize potential treatments [10].

Neutrophils are one of the first responders to sites of inflammation and play a critical role in the innate immune response. When effective, neutrophils migrate from the bloodstream through endothelial walls to the site of inflammation by sensing gradients of chemokines, which bind to neutrophil cell surface receptors. In early stages of sepsis neutrophils potentially play a duplicitous role, both actively fighting the invading pathogen but also contributing to undesirable systemic inflammation, which often leads to multiple organ dysfunction, immune paralysis, or death [11,12]. Neutrophils’ roles in sepsis are well recognized but the dynamics of multiple phenotypes and their impact on treatments is not fully understood. A key chemokine impacting neutrophil behavior and phenotype is interleukin-8 (IL-8). IL-8 signals through functionally distinct surface receptors CXCR-1/2, which are primarily expressed on neutrophils. CXCR-1 is primarily responsible for activating phospholipase D [13], which mediates respiratory burst and other pathogen killing functions. CXCR-2 has been shown to stimulate migratory functions such as chemotaxis and diapedesis [14,15].

The motivation of this work is to use computational modeling of CXCR-1/2 signaling, and the associated dynamics in neutrophil phenotype composition, to explore whether modifying this dynamic could be exploited to favorably impact outcome in sepsis. A population based mechanistic computational model, which incorporates both receptor level dynamics and neutrophil response to pathogen, was developed to explore the mechanisms involved in sepsis progression and calibrated in septic baboons. Furthermore, an experimental extracorporeal treatment which modulates CXCR-1/2 receptor levels was evaluated *in silico* using the model framework. The computational model described in this manuscript provides a physiologic rationale for neutrophil’s CXCR-1/2 mediated activity in sepsis, delivers insight into the overriding mechanisms involved, and suggests that interventions aiming to modulate phenotypic composition are time sensitive.

## Results

### Computation of Parameter Ensembles explaining Survivor and Non-Survivor dynamics

**Fig 1:**
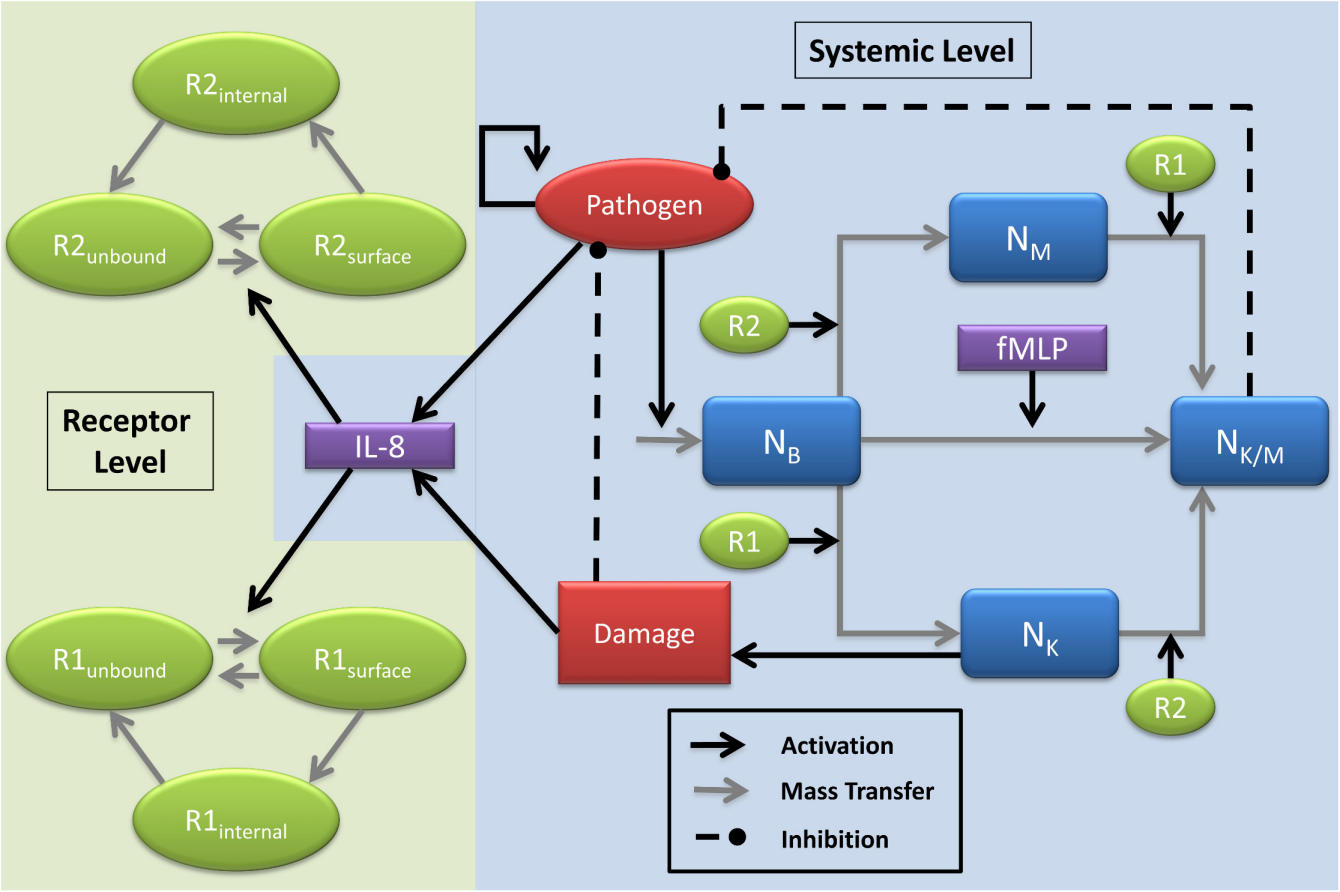
Model diagram detailing neutrophil phenotypes and critical feedback loops. The system is divided into modules based on the level at which the interactions occur. The systemic level includes the interactions between the pathogen (P), four neutrophil phenotypes (basal: N_B,_ migratory: N_M_, killing: N_K_ and killing and migratory: N_K/M_) and chemokine IL-8. The receptor level interactions include the intracellular dynamics of CXCR-1/2, namely activation, internalization and recycling. Two types of feedback occur between the two levels, active surface receptors can trigger the phenotype conversion of the neutrophils and IL-8 produced at the systemic level triggers the trafficking of the receptors. A CXCR-1/2 independent activation via fMLP is included to model general pro-inflammatory response. The systemic damage (D) indicates the overall damage (direct and indirect) caused by the action of the killer neutrophils.

Of the 16 baboons subjected to bacterial infusion, 11 (69%) died and 5 (31%) survived, with death occurring within 6 days of bacterial infusion. Based on these two systemic outcomes, a thorough investigation of the model (see Methods section & Fig 1) was completed to identify parameter regimes that explain the dynamics of each group of the responders.

The initial conditions for the state variables of the ODE were fixed to simulate experimental stimulation (Table 1). Among the rate parameters, some were fixed to literature values. These included pathogen growth and decay rates, basal decay rates of naïve neutrophils, CXCR-1/2 internalization and recycling rates and creatinine decay rate (See fixed parameters in Tables 2 and 3). Remaining parameters were estimated by generating parameter ensembles using a Bayesian parallel tempering approach that fit our model to the survivor and non-survivor experimental data sets (see Methods). We conducted the parameter estimation process in two rounds. In round one, the model was fitted to the two data sets separately. By fitting to the two data sets separately, we were able to effectively show that the model was capable of replicating both lethal and non-lethal outcomes through only a change in few parameters. In an attempt to classify the underlying differences, we identified the parameters that were most influential in determining the outcome (survivor or non-survivor) of an individual using stepwise logistic regression. This resulted in a list of seven key parameters. These parameters tend to control the rate at which neutrophils grow and how quickly they can change phenotypes, which play a critical role in determining how quickly and severely the animal will respond to the infection.

**Table 1:** Initial conditions.

No.	Symbol	Description	Initial Condition–Survivors	Initial Condition–Non-Survivors	Units
**1**	*P*	Pathogen	1000	1000	CFU
**2**	*N*_*B*_	Basal neutrophils	4.4	5.06	10^3^ cells/μl
**3**	*N*_*K*_	Neutrophils with killer phenotype	0.0	0.0	10^3^ cells/μl
**4**	*N*_*M*_	Neutrophils with migratory phenotype	0.0	0.0	10^3^ cells/μl
**5**	*N*_*K*/*M*_	Neutrophils with dual phenotype	0.0	0.0	10^3^ cells/μl
**6**	*C*_*IL*8_	Systemic IL-8 concentration	0.0	0.0	nM
**7**	*D*	Global tissue damage	0.0	0.0	unitless
**8**	*C*_*R*1*s*_	Surface CXCR1 population	0.0	0.0	unitless
**9**	*C*_*R*1*i*_	Internalized CXCR1 population	0.0	0.0	unitless
**10**	*C*_*R*2*s*_	Surface CXCR2 population	0.0	0.0	unitless
**11**	*C*_*R* 2*i*_	Internalized CXCR2 population	0.0	0.0	unitless
**12**	*C*_*creat*_	Creatinine	91.5455	102.4	nM
**13**	*F*	Filter term	0.0	0.0	unitless
**14**	*C*_*R*1*t*_	CXCR1 trapped	0.0	0.0	unitless
**15**	*C*_*fMLP*_	fMLP	0.0	0.0	nM
**16**	*C*_*elas*_	Neutrophil elastase / α1-PI complex	0.0	0.0	ng/ml
**17**	*WBC*	Total white blood cells	4.4	5.06	10^3^ cells/μl

**Table 2:** Shared parameter values.

No.	Symbol	Description	Fixed/Fitted	Mean	Std. Dev.	Units
**1**	*k*_*PG*_	Pathogen growth	Fixed	1	0	Hour^-1^
**2**	*$k_{PG-N_{K/M}}$*	Neutrophil induced pathogen death	Fitted	145.5391	0.040553	(Hour *10^3^ cells/μl)^-1^
**3**	*k*_*PL*_	Pathogen population limit	Fitted	1.6533E-7	2.3409E-7	(Hour*CFU)^-2^
**4**	*k*_*P*_	Pathogen decay	Fitted	271.9904	352.4858	CFU/Hour
**5**	*$k_{p}^{d}$*	Pathogen decay	Fixed	1000	0	CFU
**6**	*$k_{N_{B}}$*	Basal neutrophil natural	Fixed	0.1	0	Hour^-1^
**7**	***$k_{N_{K}}$***	Killer neutrophil decay	Fitted	0.0330	0.0275	Hour^-1^
**8**	*$k_{N_{M}}$*	Migratory neutrophil decay	Fitted	0.1244	0.1936	Hour^-1^
**9**	*$k_{N_{K/M}}$*	Migratory-Killer neutrophil decay	Fitted	0.1176	0.1772	Hour^-1^
**10**	*$k_{N_{M}-N_{K}-IL8}$*	IL-8 induced migratory neutrophil to neutrophil migratory-killer transition	Fitted	168.7708	260.7247	Hour^-1^
**11**	*k*_*IL*8−*P*_	Pathogen induced IL-8 production	Fitted	2.7810E-6	2.0441E-6	Hour^-1^
**12**	*k*_*IL*8−*D*_	Tissue damage induced IL-8 production	Fitted	5.7938E-9	7.9615E-9	nM*Hour^-1^
**13**	*k*_*IL*8_	IL-8 decay	Fitted	0.3352	0.0261	Hour^-1^
**14**	*$k_{D-N_{K}}$*	Tissue damage induced by killer neutrophils	Fitted	0.0319	0.0285	(Hour *10^3^ cells/μl)^-1^
**15**	*k*_*D*_	Damage recovery rate	Fitted	7.0147	8.8870	Hour^-1^
**16**	*k*_*filter*_*on*_	Filter production rate	Fitted	6.1698E-4	8.2047E-4	(CFU*Hour)^-1^
**17**	*k*_*r*1_	Dissociation constant for R1 receptors	Fixed	79.2	0	Hour^-1^
**18**	*k*_*r*2_	Dissociation constant for R2 receptors	Fixed	79.2	0	Hour^-1^
**19**	*k*_*D*_	Affinity constant for IL-8 to the receptors	Fixed	2.5E-3	0	Hour^-1^
**20**	*k*_*i*1_	Internalization rate for IL-8-R1 complex	Fixed	5.196	0	Hour^-1^
**21**	*$k_{i1^{'}}$*	Recycle rate for R1	Fixed	0.612	0	Hour^-1^
**22**	*k*_*i*2_	Internalization rate for IL-8-R2 complex	Fixed	20.796	0	Hour^-1^
**23**	*$k_{i2^{'}}$*	Recycle rate for R2	Fixed	0.144	0	Hour^-1^
**24**	*k*_*fMLP*_	Pathogen induced fMLP production	Fitted	5.9866E-7	1.3864E-6	nM*Hour^-1^
**25**	*$k_{fMLP}^{d}$*	Pathogen induced fMLP production	Fitted	622.9280	1.6952E3	CFU
**26**	*k*_*fMLP*−*D*_	Pathogen induced fMLP decay	Fitted	9.8425E4	1.7303E5	Hour^-1^
**27**	*$k_{fMLP-N_{B}}$*	fMLP induced basal neutrophil to migratory-killer phenotype transition	Fitted	0.0021	0.0025	Hour^-1^
**28**	*k*_*ne*_	Scaling of Nk cells to neutrophil elastase / α1-PI complex levels	Fitted	0.0351	0.0209	ng/cell

**Table 3:** Unique parameter values.

No.	Symbol	Description	Fixed/ Fitted	Mean—Survivor	Std. Dev.–Survivor	Mean–Non-survivor	Std. Dev.–Non-survivor	Units
**1**	*k*_*NG*_	Neutrophil baseline growth rate, based on 12 h life	Fixed	0.506	0	0.54417	0	10^3^ cells/ (μl *Hour)
**2**	*k*_*creat*_	Creatinine decay rate	Fixed	0.1591	0	0.1792	0	Hour^-1^
**3**	*$k_{N_{B}-G}$*	Pathogen influenced neutrophil growth	Fitted	6.6447E5	1.5092E6	6.4953E5	1.0063E6	unitless
**4**	***$k_{N_{B}-G}^{d}$***	Pathogen influenced neutrophil growth (denominator)	Fitted	8.8322E4	1.7149E5	5.5590E4	9.0741E4	unitless
**5**	*$k_{N_{K}-IL8}$*	IL-8 induced neutrophil basal to killer phenotype transition	Fitted	8.9617E3	4.9596E3	4.5302E4	2.6386E4	Hour^-1^
**6**	*$k_{N_{M}-IL8}$*	IL-8 induced neutrophil basal to migratory phenotype transition	Fitted	4.8740E3	6.5289E3	2.9804E3	6.6655E3	Hour^-1^
**7**	*$k_{N_{K}-N_{M}-IL8}$*	IL-8 induced killer neutrophil to neutrophil migratory-killer transition	Fitted	3.9436	12.6137	5.9631	16.7691	Hour^-1^
**8**	*$k_{IL8-P}^{d}$*	Pathogen induced IL-8 production	Fitted	1.2814E4	1.2646E4	1.2841E4	1.4198E4	CFU
**9**	*k*_*filter*_*off*_	Filter decay rate	Fitted	0.0813	0.0145	0.1407	0.0328	Hour^-1^

**Fig 2:**
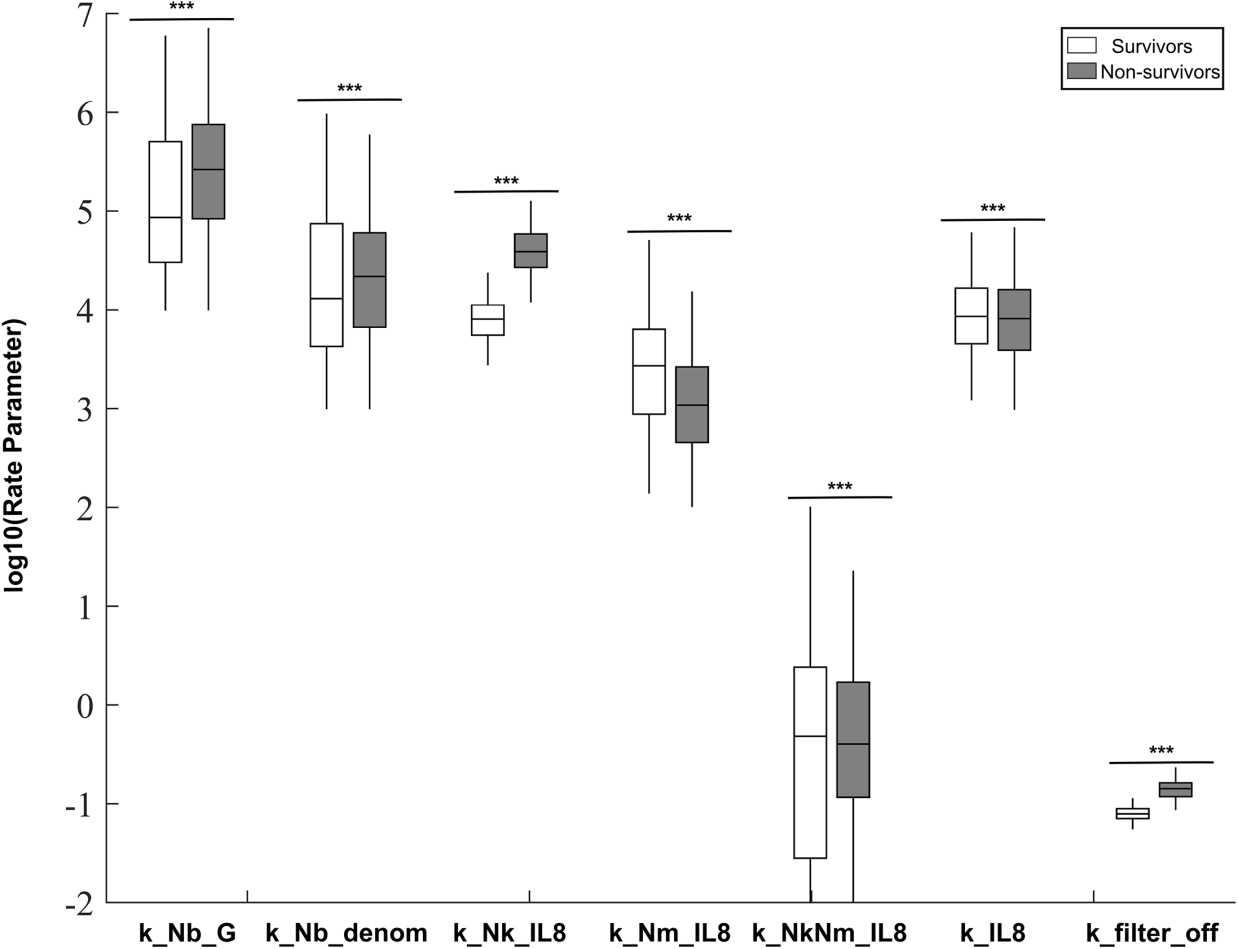
Posterior distributions of parameters allowed to vary across ensembles. Each parameter was fit separately to data from surviving and non-surviving animals. Values for the mean, 25^th^-75^th^ percentile, and 2.5^th^ to 97.5^th^ percentiles are shown. Parameters distributions were compared using a two-sample Kolmogorov-Smirnov test. *p<0.05, **p<0.01, ***p<0.001.

Once these differentiating parameters were identified, we put the model through a second round of estimation. In the second round, the model was fit to both data sets simultaneously; allowing only the seven previously identified key parameters to vary between the survivor and non-survivor subpopulations (see Table 3). Additionally, two fixed parameters were allowed to take different values across the two populations to maintain the appropriate initial conditions in creatinine and white blood cell count. This step resulted in two new parameter ensembles that were identical in 28 parameters but varied in nine parameters. This second step enabled us to better crystallize the differences between animals that survived and those that died. These ensembles are biologically more relevant as we expect the animals’ immune responses to be highly similar, with small but important differences indicating susceptibility to a septic insult. Resulting full marginal distributions for each of the 7 parameters were statistically different across survivor and non-survivor populations (Fig 2). The final mean values and the standard deviation of all the estimated parameters are summarized in Tables 2 and 3.

### Features of Survivor and Non-Survivor dynamics

#### Trained model outcomes

**Fig 3:**
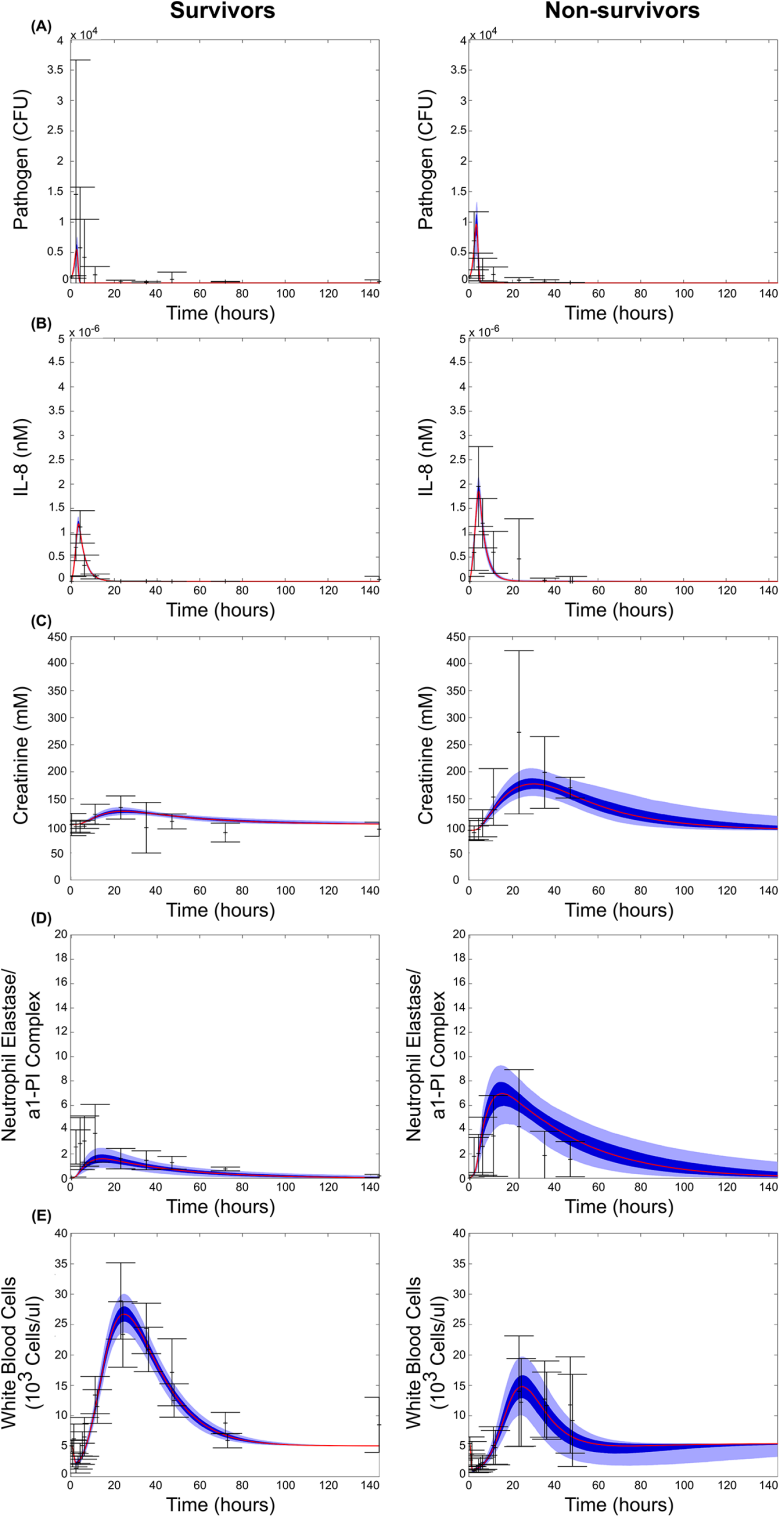
Simulated model fits with their experimental training data. Mean (red), 25^th^-75^th^ percentile (dark blue), and 5^th^-95^th^ percentile trajectories of the simulated ensemble are shown. Experimental data points are shown in black with error bars representing one standard deviation above and below the mean. Results are shown for surviving (left) and non-surviving (right) animals for all observables with corresponding experimental data; **(A)** pathogen levels, **(B)** free IL-8 levels, **(C)** white blood cell counts, **(D)** neutrophil elastase / α1-PI complex levels, and **(E)** creatinine levels.

The two ensembles resulted in model fits that faithfully recreate the key features of the surviving and non-surviving data sets (Fig 3). Pathogen dynamics showed a transient behavior, with the model predicting a slightly higher peak for non-survivors. The ensembles captured the transient peak in IL-8 that occurs early after infection, with the non-surviving population exhibiting a higher maximum peak. The predicted neutrophil populations also tracked well with the experimental results, with circulating basal neutrophils exhibiting a strong initial decline in abundance as the cells are activated and migrate to the site of infection, followed by a growth phase as the body compensates for the infection, and finally a return to baseline levels. While both surviving and non-surviving populations exhibited this trend, the surviving populations had a noticeably higher peak in basal neutrophils during the growth phase. Levels of neutrophil elastase / α1-PI complex in the blood, indicative of the killing and damage causing function of activated neutrophils, peaks around 15 hours post-infection. The non-surviving population showed a stronger and longer lasting peak, which is captured by the model. Creatinine, a measure of kidney health, increased to higher and more sustained levels in non-surviving animals, as kidney health decreases and creatinine was not as efficiently cleared.

#### Model predictions

**Fig 4:**
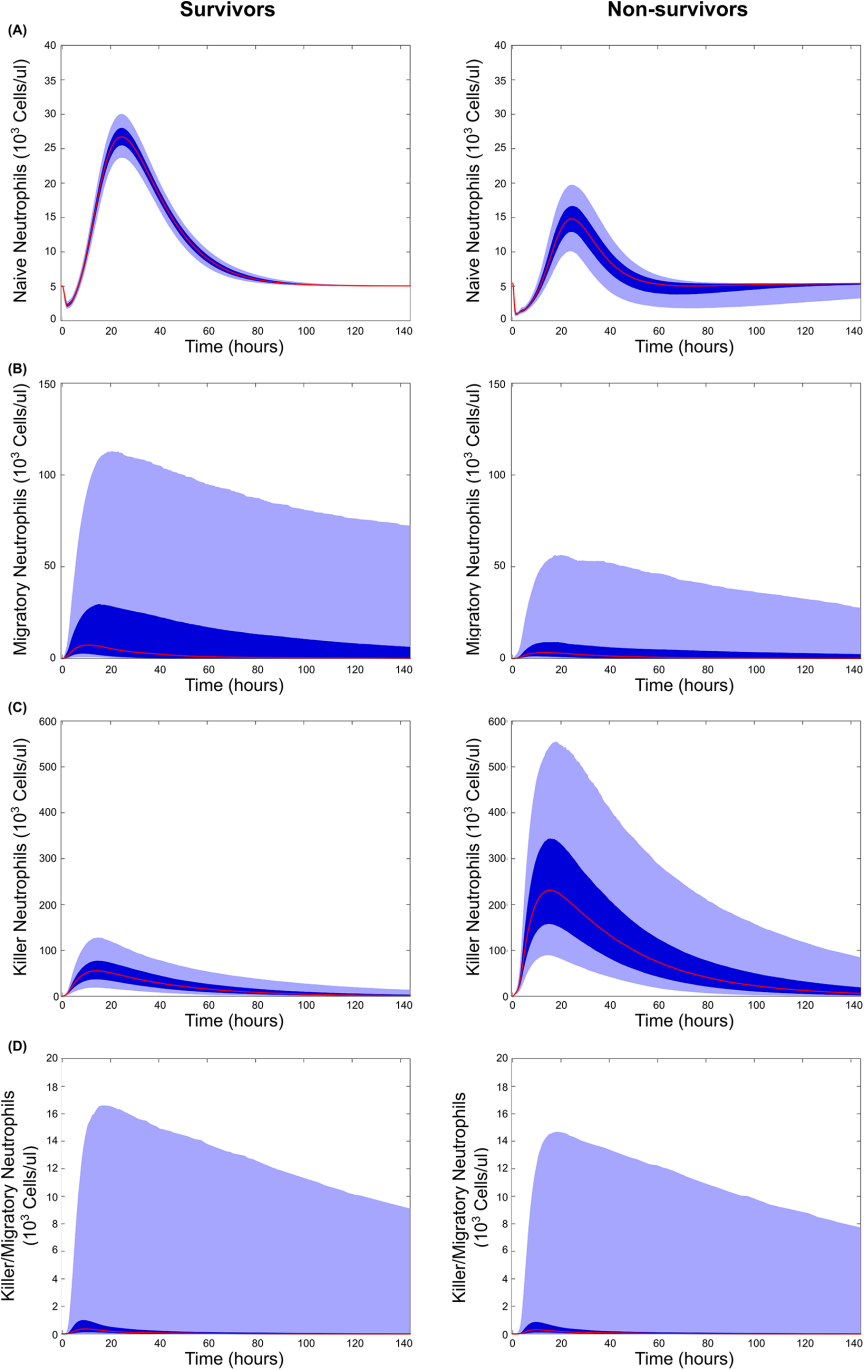
Model predictions for neutrophil phenotype dynamics following infection. Mean (red), 25^th^-75^th^ percentile (dark blue), and 5^th^-95^th^ percentile trajectories of the simulated ensemble are shown. Predictions are shown for surviving (left) and non-surviving (right) animals for the four neutrophil phenotypes considered in the model; **(A)** basal neutrophils, which were calibrated with white blood cell count data, as well as **(B)** migratory neutrophils, **(C)** killer neutrophils, and **(D)** killer/migratory neutrophils for which there is no experimental data.

**Fig 5:**
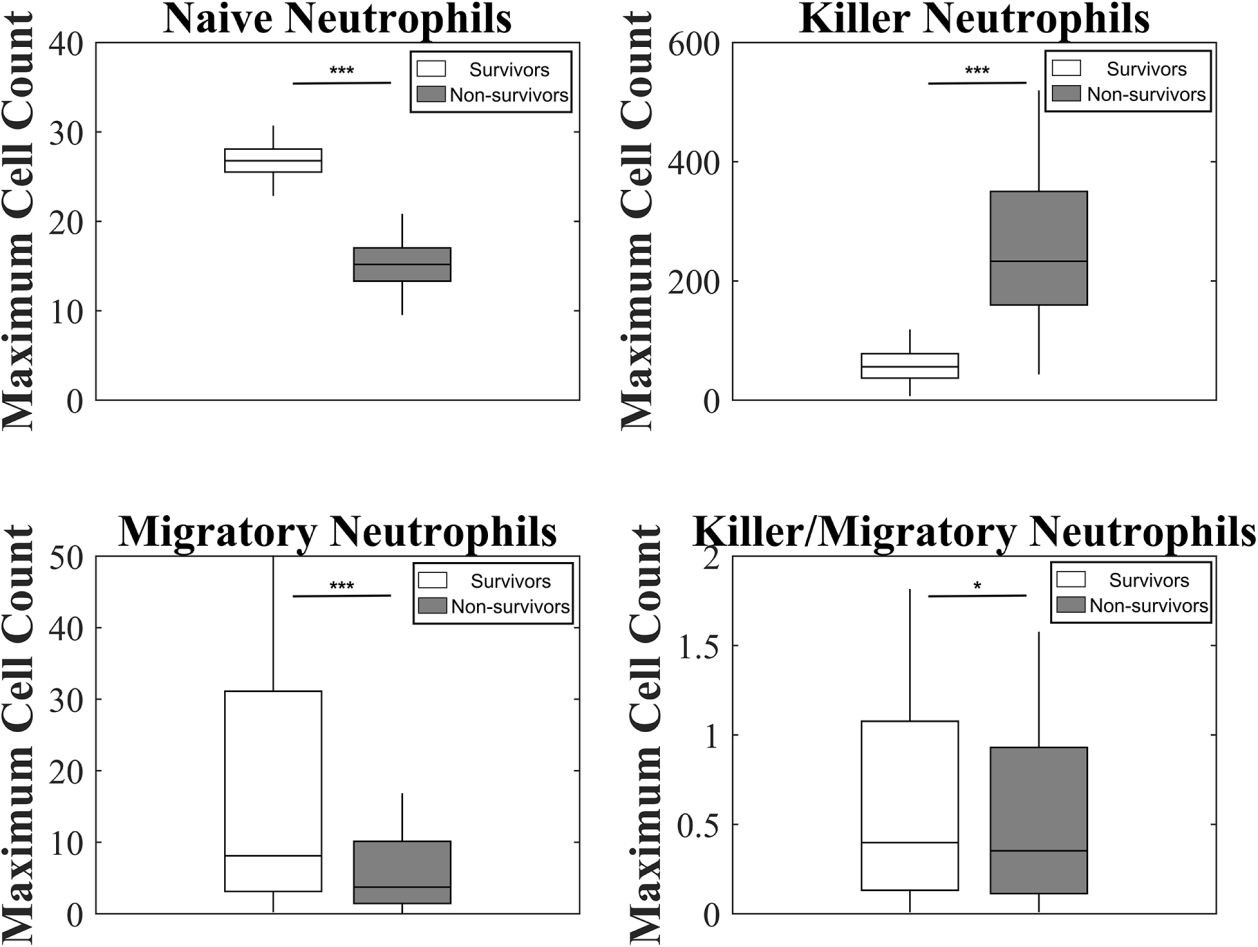
Model predictions for maximal levels of each neutrophil phenotype compared across ensembles. Maximal values for each neutrophil phenotype from each trajectory in both ensembles were recorded. Values for the mean, 25^th^-75^th^ percentile, and 2.5^th^ to 97.5^th^ percentiles are shown. Distributions were compared using a two sample T-test. *p<0.05, **p<0.01, ***p<0.001.

The model also made predictions in the absence of observable data on the dynamics of neutrophil phenotypes (Fig 4 & Fig 5). Although both populations had similar peaks in fully activated neutrophils, allowing them both to fight off the infection on similar time scales as predicted experimentally, they showed strong differences in other populations. Non-survivors showed a significantly stronger spike in damage-causing killer neutrophils, while survivors showed a stronger spike in migratory neutrophils. This can also be seen in the parameter ensembles, as neutrophils in non-survivors had an increased proclivity to activate their killing function in response to IL-8, while neutrophils in survivors were faster to activate their migratory functions (Fig 2). Generating similar numbers of fully activated neutrophils, but through differing intermediate activation populations, could be an explanation for how these two animal populations controlled infection with similar dynamics, while still experiencing differing fates.

At the receptor level, underlying activation of CXCR-1/2 was transient in both the survivors and non-survivors (S1 Fig). Compared to the neutrophil dynamics which was slow and spread across few hours, receptor dynamics was very fast. Most of the receptors were in the free state, and internalized CXCR-1 is recycled faster than CXCR-2. Among the active receptors, there was one order of magnitude higher level of internalized CXCR-1 receptors than the surface bound CXCR-1 receptors, while this difference is two orders of magnitude for CXCR-2. Non-survivors had higher levels of the surface and internalized active receptors. This can be explained by the higher peak in IL-8 levels for the non-survivors than the survivors (Fig 3). But, survivors had very close levels of CXCR-1 and -2 bound receptors and non-survivors had slightly higher levels of bound CXCR-1 than bound CXCR-2. These small differences in the peak levels of the receptors coupled with differences in the transition rates were sufficient to result in different neutrophil phenotype levels in the two populations, a key prediction from the ensemble modeling process.

### Factors modulating cumulative damage in the two populations

**Fig 6:**
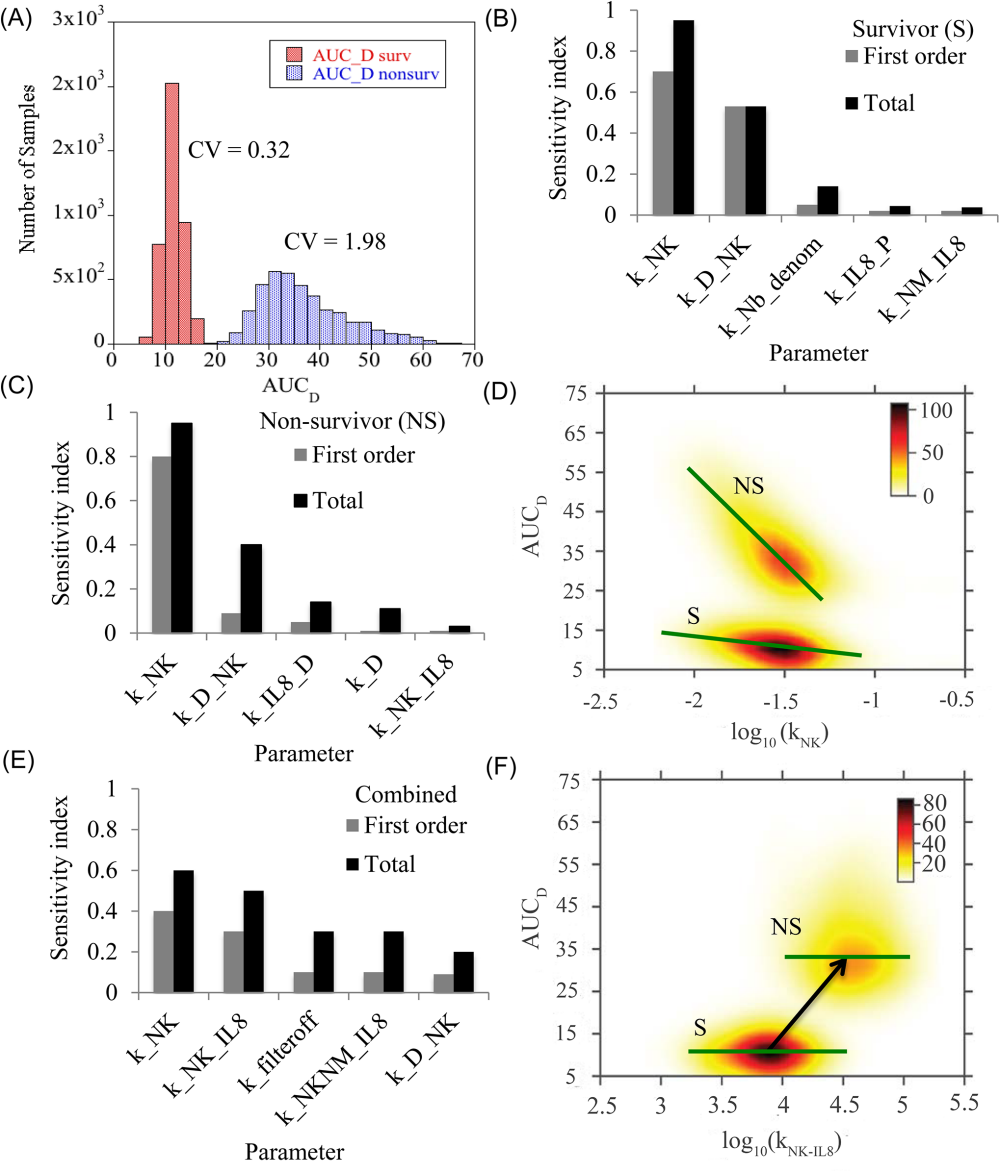
Factors affecting cumulative systemic damage. **(A)** Cumulative damage seen in survivors and non-survivors. The histograms show the area under the damage curve until 144 hr. The rate parameters were sampled from the generated ensemble for each population. The distribution used for GSA contains 4000 samples for each population. **(B-C)** Prime drivers of cumulative damage. First order and total effect Sobol’ indices which explained most of the variance are tabulated here for the survivor and non-survivor population respectively. **(D)** Functional dependence of AUC_D_ on killer cell decay rate for the survivors (S) and non-survivors (NS). The green line has been added for visual guidance of the trend and is based on the mean trend identified by the RS-HDMR component functions. For each population, damage decreases with increase in the decay rate of the killer neutrophil. **(E)** Prime drivers of cumulative damage for the combined population. **(F)** Functional dependence of AUC_D_ on CXCR1 induced naïve to killer neutrophil transition rate for the survivors and non-survivors. The green line shows that within the population, damage is not particularly sensitive to the transition rate, but increased transition rate could be responsible for higher damage levels seen in non-surviving population.

Until now, the focus was on deriving parametric ensembles explaining the mechanism of sepsis progression in each population. In this section, the sensitivity of sepsis-mediated damage to different model parameters (and hence different processes in the network) was evaluated for each population. Area under the damage curve (AUC_D_) was used as an output metric of cumulative damage from sepsis. The analysis was done in two steps. First the sensitive parameters affecting damage in each population was identified to check if similar parameters were responsible for modulating damage within each population. Next, the two populations were combined to identify the parameters primarily responsible for a switch from a low to a high damage region. Since the model is highly nonlinear, a global sensitivity analysis (GSA) based on variance decomposition was chosen. This method decomposes the total variance in the output into variance and co-variance contributions from each rate parameter and its higher order combinations. To reduce computational cost, a meta-model based approximation was done (See Materials and Methods). The meta-model method called Random Sampling High Dimensional Model Representation (or RS-HDMR), decomposes the output function (AUC_D_) into a set of component functions that includes the mean followed by first order effects of each parameter and other higher order effects resulting from parameter combinations. The degree of sensitivity of a parameter or its combination with other parameters (as a set) is captured by Sobol’ index which by definition is the fraction of the total output variance attributed to the selected parameter set. To perform GSA, 4000 samples were generated from the parameter distributions of the two ensembles and the dynamics of the damage term was simulated for the survivors and the non-survivors. Fig 6(A) shows the AUC_D_ distributions for each ensemble. As expected, the survivors show lower levels of cumulative damage than the non-survivors. The coefficient of variation was higher for the non-survivors (CV = 1.98) as compared to the survivors (CV = 0.32). When GSA was performed on the survivor and non-survivor samples separately and in combination, it was found that a third order RS-HDMR contributed close to 95% of the variance for both the populations. However, most of the important contributions were from the parameters constituting highly ranked first order indices. Fig 6B and 6C shows the first order and total Sobol’ indices for the first five most sensitive parameters of each population and Fig 6(E) shows the results when both populations are combined. Note that the total Sobol’ index for each parameter, is the sum of first order index and all higher order indices involving that parameter.

For GSA conducted separately on the survivor and non-survivor ensembles, it is found that damage is mainly determined by the decay rate of the killer neutrophils, $k_{N_{K}}$ (direction of influence shown in Fig (6D)). The decay rate of the killer neutrophil controls the rate at which killer neutrophils are removed from the system, and the faster these neutrophils are removed, the lesser the damage. The next important term is the direct damaging effect of the killer neutrophils and this parameter has significant second order interactions with other parameters of the model as seen from the total sensitivity index. The next set of parameters has secondary importance and these parameters are different for the two populations (variance contributions of each parameter in this set is in the range, 1–10%). In survivors, damage is more influenced by the production rate of basal neutrophils and IL-8 in presence of the pathogen. In non-survivors, the effect is more pronounced for damage mediated IL-8 production (a positive feedback component), damage recovery term and killer neutrophil production rate. This indicates that overall damage in non-survivors is more sensitive to the parameters associated with killer cells, IL-8 and damage.

For GSA conducted on the combined population, the decay rate of killer neutrophils remains the most important parameter. Interestingly, the sensitivity value and ranking of three parameters increase relative to the case where the populations are analyzed separately. Among these, the transition rate of naïve neutrophils to the killer phenotype via CXCR1 ($k_{N_{K}-IL8}$) is the most important parameter. The next two parameters include the decay rate in filter Eq (7) (which determines the delay between pathogen generation and resulting neutrophil entry into circulation during sepsis) followed by the parameter controlling transition rate of killer neutrophil to the dual phenotype by CXCR2. Functional dependence of damage on these three parameters shows that they could be responsible for shift in the population from a low to a high damage region. For example, Fig 6(F) shows the dependence of AUC_D_ on parameter $k_{N_{K}-IL8}$. Within each population, no particular trend is visible, but relative increase in the transition rate in the non-survivors correlates well with increased damage. Results in Fig 2 showed that the ranges of two of the parameters, $k_{N_{K}-IL8}$ and *k*_*filter*_*off*_ were significantly different for the survivors and non-survivors. Results from sensitivity analysis support this prediction and further show that the parameter values correlate well with the transition in observed damage.

### Treatment implementation

**Fig 7:**
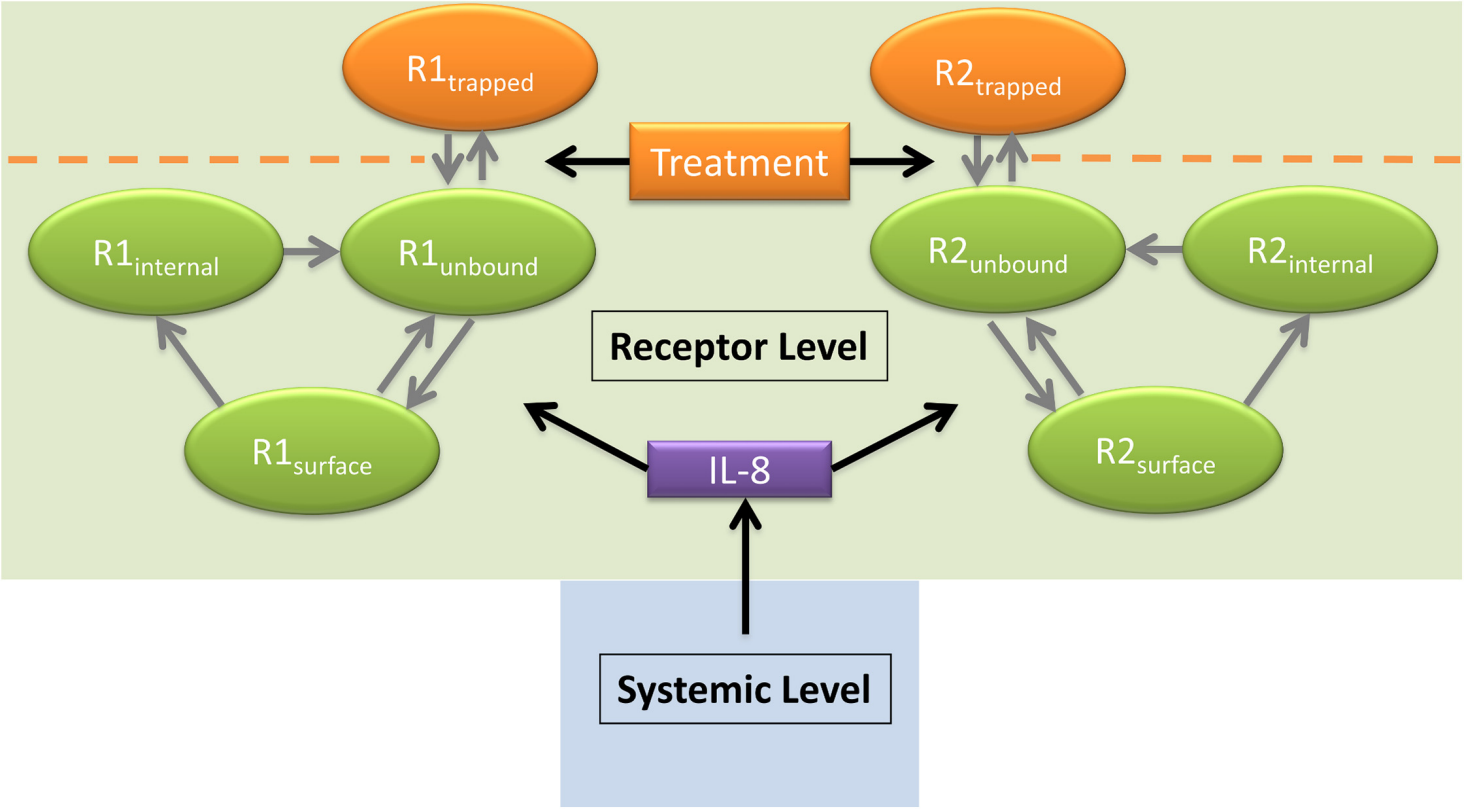
Model diagram showing receptor level treatment implementation. The extracorporeal treatment introduces a trapped receptor state for CXCR-1/2. This state prevents IL-8 induced phenotype transition, which limits N_K_ generation. The treatment is modeled entirely in the receptor level of model, leaving the systemic level (see Fig 1) unchanged.

Extracorporeal devices are emerging as promising therapies for treatment of sepsis[16–19]. In this instance we propose extracorporeal treatment which directly modulates CXCR-1/2 levels using a bioactive surface which interacts with unbound neutrophil surface receptors upon contact. Such a device, which is currently under development at the University of Pittsburgh, generates targeted and controlled downregulation of neutrophil surface receptors. The dynamics of this device can be analyzed within the framework of the generated computational model to determine its proof of principle *in silico* and help optimize treatment parameters. The proposed treatment implementation is shown in Fig 7. Specifically, the receptors are allowed to go to a trapped state and become unavailable for activation by IL-8 for the indicated time of treatment. To evaluate the potential of such an immunomodulatory treatment, we next performed an *in silico* trial by varying (1) the time when the treatment is introduced and removed and (2) the strength of interaction between the trapping device and the unbound neutrophil surface receptors.

#### Impact of treatment parameters

For the analysis, the treatment initiation time was varied between 0 and 12 hours after the initial infection and the treatment discontinuation time was varied between 0 and 100 hours after infection. To modulate the treatment intensity, the device-receptor K_d_ was varied between the 1x10^-2^ M and 1x10^-5^ M, with 2.5e-3 M representing the K_d_ of IL-8 and the receptors. The treatment was tested on a simulated population constructed by randomly selecting 69% of parameter sets from the non-survivor ensemble and 31% of parameter sets from the survivor ensemble, as observed in the experimental population. Survivor rate was measured for each set of proposed treatment parameters, as determined by the logistic regression classifier trained on the parameter ensembles with no treatment. A survivor rate above 31% was considered an improvement over baseline, and below 31% indicated the treatment causing overall harm.

**Fig 8:**
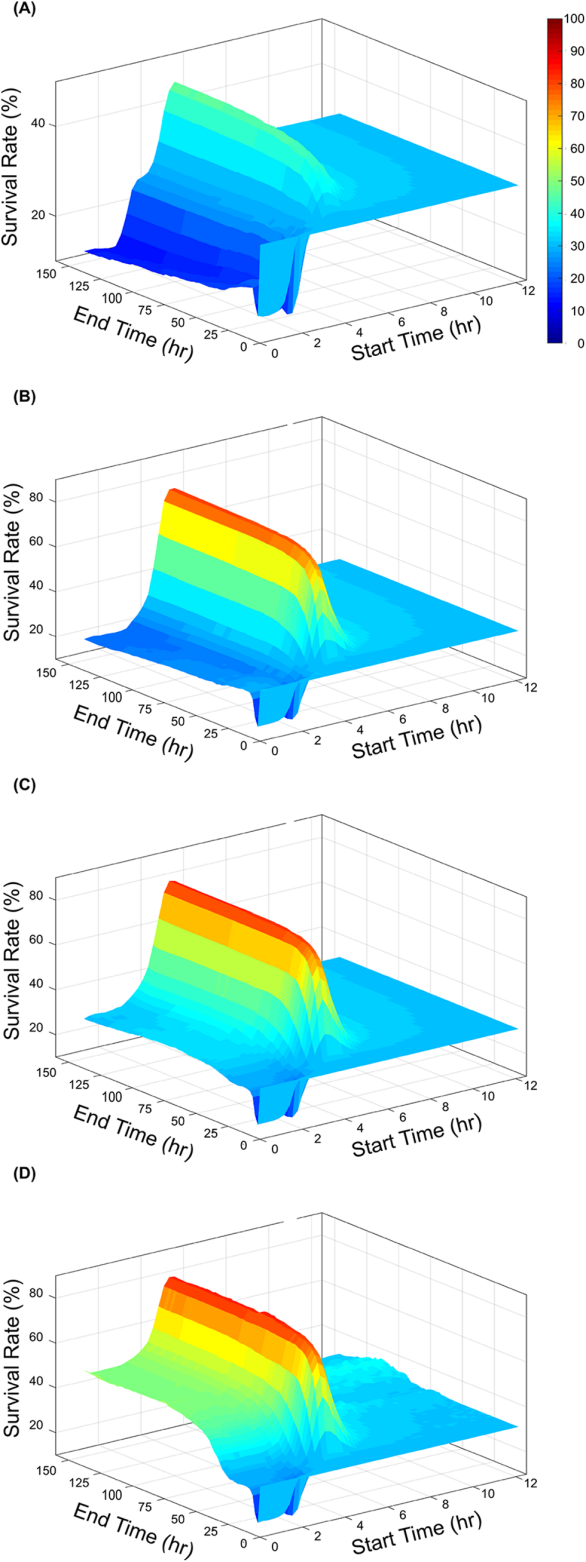
Effects of simulated treatment on animal survival rates. Survival rates of a simulated population of animals following treatment with the proposed extracorporeal device considering a device-receptor affinity of **(A)** 1x10^-2^ M, **(B)** 1x10^-3^ M, **(C)** 1x10^-4^ M, **(D)** 1x10^-5^ M. In all cases the time of treatment was varied between 0 and 12 hours post infection and ended between 0 and 100 hours post infection.

Fig 8 shows the survival rate following different treatment strengths and start-end times. In general, the optimal time for beginning treatment was between 3 and 6 hours after the original infection, resulting 40–80% survival rates depending on treatment strength. Starting the treatment after six hours was typically too late to have a strong effect on survival. Starting treatment within 3 hours of infection would often have neutral or deleterious effects, as it would dampen the initial inflammatory response that is critical to fighting off the infection. This led to an increase in pathogen growth and an increased late inflammatory response once treatment was removed. In the worst case scenarios following early treatment of a short duration, survival rates dipped as low as 13.2%, and this trend could be seen across all treatment strengths.

When treated at the optimal time, survival rates increased from the 31% baseline to greater than 80% with sufficient device-receptor affinity. Using a K_d_ of 1e-2 M results in a maximum survival rate of 47%, and decreasing the K_d_ to 1e-3 M further increases this rate to 80.3%. Further decreases in the K_d_ to 1e-4 M and 1e-5 M results in increases in survival rate to 83.1% and 84.4%, showing there is a diminishing return to continuously increasing the device affinity. As the affinity increases, we see a new trend emerge in the simulation results, where treatment that begins as early as the onset of infection and is significantly long lasting leads to increased survival rates, and a less strictly defined optimal treatment time (Fig 8(D)). In this case, the treatment is so strong and long-lasting that the inflammatory response is very strongly suppressed, implying that overwhelming pathogen growth leading to death cannot be reached within the bounds of this. However, this suppression of the immune system allows for significant pathogen growth and could leave the subject vulnerable to secondary infections which are not considered in this model.

Trends in response to treatment also appear to be robust to individual parameter values. The two most sensitive parameters $k_{N_{K}}$ and $k_{N_{K}-IL8}$ were varied, increasing and decreasing each by 10% and 50% and recalculated the simulated population response to treatment (S2 Fig and S3 Fig). In general response trends remained the same, with a defined peak in survivorship when treatment is administered 2–4 hours after infection. The magnitude of responses varied predictably, as strongly increasing $k_{N_{K}}$, the death rate of damage-causing neutrophils, resulted in a higher peak of survival. Conversely, increasing $k_{N_{K}-IL8}$, which corresponds to a faster induction of damaging-causing cells, leads to a slight decrease in survivorship. Varying $k_{N_{K}}$ had a larger effect on these results, as expected following its identification as the model’s most sensitive parameter affecting damage (Fig 6).

## Discussion

This manuscript discusses the development of a mechanistic computational model of IL-8 mediated activation of CXCR-1/2 receptors in baboons which were administered intravenous *E*. *coli*. Neutrophil phenotypes, which dictate neutrophil functional response, were generated *in silico* based on CXCR-1/2 surface receptor levels, linking receptor level dynamics with neutrophil functional response. Parameter ensembles were generated for survivor and non-survivor populations, allowing for *in silico* observation of sepsis progression. Additionally, an extracorporeal treatment which modulates CXCR-1/2 levels on neutrophils was introduced *in silico*. This proof of concept evaluation allowed for preliminary device evaluation and optimization of treatment parameters.

To our knowledge, this is the first model describing dynamic interactions of neutrophils which specifically takes into account information sharing between the systemic variables and the receptor levels. The receptor level dynamics of the model function on a rapid time scale, adjusting to systemic IL-8 levels in a matter of minutes. These changes in receptor signaling dictate changes in neutrophil phenotype, which dictates neutrophil function and hence mortality. This link thus provides a valuable mechanistic framework that can be subjected to clinically relevant treatment scenarios. For example, the experimental treatment could be implemented purely on the receptor level. Alternatively, systemic variables such as IL-8 levels or neutrophil phenotype could be modulated to evaluate performance of hemoadsorption or neutrophil sequestration extracorporeal devices.

Application of parallel tempering approach for parameter estimation allowed for the efficient generation of ensembles of parameters and resulted in a model that could fit experimental data well [20], allowing reasonably accurate simulations of the system without making strong claims about the values of single parameters which are notoriously difficult to measure and are likely to vary between individuals. This allows for robust, population-level predictions rather than point predictions of model parameters and model behavior. However, the computed multi-dimensional posterior distribution in parameter space reflects constraints imposed by empirical data, as well as data sparsity and uncertainty. These constraints impose a covariance structure in the posterior distribution such that there is robustness in model behavior, despite large uncertainties in individual parameter values. Learning this structure is likely crucial in building predictive model [21,22]. Yet, the method is making no claim that individual parameter sets in the ensemble represent individuals in a population. At best, an individual could be represented by a smaller ensemble, reflecting uncertainty relating to this particular individual. Yet, it is fair to say that the ensemble is meant to represent uncertainly about a population of individuals, so that simulating the ensemble will provide expected behaviors across a population of individuals, as long as such behaviors are compatible with the empirical data used to generate the ensemble.

One trend that arose in the estimated parameter ensembles was a large difference in the magnitudes of different rate constants, sometimes spanning many orders of magnitude. This is not surprising, due to the inclusion of biological events spanning many time scales, ranging from fast molecular events to cell phenotype transitions and finally to the full duration of infections lasting for days. This suggests that future iterations of the model would benefit from a multiscale approach optimized towards handling these different time scales. Previous efforts [23–25] have worked out approaches that allow for efficient deterministic simulation of fast-scale molecular events, combined with more accurate stochastic simulation of slow-scale or rare events, and such techniques have resulted in impressive results [26,27].

Sensitivity analysis on the parametric ensembles enabled identification of the relative importance of the model parameters to state variables of the model. In general, sensitivity analysis is an important step in systems biology workflows and provides valuable information on model characteristics [28,29]. Most models in the literature resort to a local analysis which is sufficient if the parameters are well defined. For nonlinear dynamic models based on sparse experimental data and for systems which have inherently high parametric uncertainty, a global analysis needs to be done. Global techniques perform combinatorial perturbations of the parameters utilizing samples from the high-dimensional space. Application of meta-modeling approximations via RS-HDMR as was done in this work can significantly reduce the computational cost of sampling requirements for global methods. Additionally, if the sampling process takes into account parameter covariance computed from an ensemble model, biologically relevant sensitivity indices can be obtained. The systematic integration of ensemble modeling and global sensitivity analysis in this work allowed for identification of the parameters that control biological outcomes like sepsis induced tissue damage.

In addition to parameter fits, the behavior of the non-fitted state variables were inspected to check for features relevant to a clinical prognosis. Sepsis progression was analyzed by comparing differences between survivor and non-survivor populations. Neutrophil phenotypes in particular give insight into the differences between survivors and non-survivors. Of importance is the killer neutrophil population, which is highly elevated in the non-survivor population (see Figs 4 & 5). This neutrophil phenotype is associated with neutrophil induced tissue damage in the model. With support from sensitivity analysis, killer neutrophil decay rate, which sets the levels and dynamics of *N*_K_, was found to be the most important contributor to total damage in both the populations. Multiple studies support this finding, indicating that non-survivors or those with more severe sepsis experience increased levels of neutrophil induced tissue damage and MPO generation [11,30–33]. Furthermore, the importance of this term is supported by studies on neutrophil apoptosis and lifespan. Research by Taneja [34] and Fialkow [35] determined that neutrophil apoptosis was reduced in cases of severe sepsis, leading to increased lifespan of primed and activated neutrophils. Damage caused by these neutrophils was partially responsible for the progression of sepsis in these severe cases. Upon completion of the combined GSA, $k_{N_{K}-IL8}$ was also found to be a significant contributor to total damage. Increase of this term leads to preferential generation of the *N*_K_ neutrophil phenotype, which directly contributes to tissue damage.

On the other hand neutrophils in the migratory phenotype were similar in survivor and non-survivor populations. These findings agree with the data from Cummings *et al* [32] which found neutrophil’s harvested from septic and non-septic patients migrated to IL-8 at similar levels. Interestingly, survivors and non-survivors had similar levels of neutrophil kill/migrate phenotype, indicating that both ensembles had adequate neutrophil populations to eliminate the source pathogen. Therefore, the additional damage in non-survivors was neutrophil induced resulting from elevated neutrophil killer phenotype levels. The IL-8 mediated killing functions of neutrophils are primarily triggered through CXCR-1 rather than CXCR-2. Modulation of CXCR-1 levels in particular may reduce the killing neutrophil phenotype and reduce neutrophil induced organ damage.

A number of experimental treatments for sepsis and other acute inflammatory diseases have targeted the CXCR-1 receptor with success in animal models [36–38]. However, translation to humans has been difficult for two main reasons [6]. First are inherent species dependent differences between human and animal immune systems that must be recognized and accounted for in pre-clinical studies. Second is the misuse of animal models and misinterpretation of pre-clinical data [39]. The recent debate on the translational fidelity of critical disease mouse models is a prime example where two separate comparisons of the human versus mouse genomic leukocyte responses using the same database resulted in two contradictory conclusions [40,41]. In the case of IL-8 signaling, which is not present in murine models, homologous cytokines and their associated surface receptors must be examined in IL-8’s place [42]. In this context, in silico modeling is an attractive alternative given that it allows preliminary evaluation of experimental human treatments at minimal costs.

Multiple extracorporeal sepsis treatments are currently under investigation with promising results. Blood purification techniques such as hemoadsorption [16,17,43–45] allow for cytokines and other detrimental proteins to be removed directly from the blood during the cytokine storm, curbing the patient’s immune response. Another approach called activated neutrophil sequestration [19,46], selectively removes harmful neutrophil phenotypes from circulation. In this instance we propose extracorporeal treatment which directly modulates CXCR-1/2 levels using a bioactive surface which interacts with unbound neutrophil surface receptors upon contact, resulting in CXCR-1/2 downregulation. This approach is advantageous because no components of blood are removed from circulation, allowing for a healthy immune response after appropriate modulation of neutrophil surface receptors. In addition, all necessary cell-cell interactions are allowed to occur within well-controlled microcirculation of the device. Such a setup also allows treatment to be easily titrated or halted by adjusting blood flow through the device. The dynamics of such a device were analyzed within the framework of the generated ensemble model to determine its proof of principle *in silico* and to evaluate its benefits in rescuing individuals marked as non-survivors by the parameter ensembles.

When evaluated *in silico* the proposed extracorporeal CXCR-1/2 modulation device improved mortality from 31% to above 80% when deployed under certain ranges of conditions. This substantial improvement in survival supports the hypothesis that a CXCR-1/2 modulatory device may improve patient outcomes. However, time and length of treatment implementation are critical parameters tied to this success. The importance of quickly beginning sepsis treatment has been well established [47], particularly for antibiotic administration. Our simulations showed a well-defined optimal time for the initiation of treatment, between 3 and 6 hours after the onset of severe infection. Treatment, if started within this time frame, had a high degree of success over a large range of treatment durations and strengths. This window is specific to the animal model under study and will not directly translate to a clinical setting for two main reasons. First, the model was calibrated with experimental data obtained from baboons, and differences between the baboon and human immune systems must be considered. Second, the baboons were exposed to a well-controlled bacterial infusion at a known time point, followed by a predictably quick and strong immune response. In this instance the pathogen load is well controlled and a large portion of the ensemble can therefore be addressed by a single treatment setting. In clinical practice, patients present with varied pathogen loads and they may be in different stages of infection and immune response. So, future experiments will need to combine clinical knowledge with additional data gathering and simulation to obtain treatment timing relevant for human patients.

Clinicians are actively searching for biomarkers to track sepsis disease progression and prescribe treatment [48–50]. Neutrophil phenotype may be a valuable indicator of disease state and individual patient response, but this information is difficult to collect in the clinic. Currently neutrophil phenotype can be evaluated either through functional testing or flow cytometry analysis of critical neutrophil surface receptors. In addition to CXCR-1/2 which are the focus of this model, CD11b, CD88, and CD62L all have roles in dictating neutrophil phenotype [51] and surface receptor expressions vary depending on severity of the inflammatory response. To more readily exploit phenotype data it may be possible to map neutrophil function to easily measurable biomarkers. Using these indirect measures of neutrophil phenotype can guide clinicians to ideal treatment regimens.

In conclusion, the ensemble model presented in this report provided key insights into the progression and mechanisms involved in progression of sepsis. We underline the role of relative abundance of killer, migratory and dual neutrophil phenotypes in deciding survivorship in an animal model. In addition, an *in silico* extracorporeal treatment which modulates CXCR-1/2 neutrophil surface receptors showed promising results. Further study and collection of experimental data will help further refine both the model and experimental device. Incorporation of data from a diverse patient population and expansion of current ensembles would increase the model’s generalizability, improving the potential for translation. Additional model parameters related to the device such as flow rate, surface area, and form factor could be included, allowing the model to streamline device development.

## Methods

### Experimental data set protocol

After general anesthesia, instrumentation and a 30 minute stabilization period, sixteen baboons (*Papio ursinus*) weighing between 19 and 32 kg were infused with 2 x 109 CFU *Escherichia coli* per kg over a two-hour period as described previously [52]. Thereafter, antibiotic therapy was delivered (gentamycin 4mg/kg twice a day). Eight animals were placed in an acute study lasting 6 days, while another eight were placed in the chronic study intended to last 28 days. All animals were observed for a 4-hour period after bacteria infusion then 11, 23, 35, 47, 72 hour and 6 days after infusion. Pathogen counts in blood, IL-8, creatinine, white blood cell, neutrophil elastase / α1-PI complex, and other physiologic parameters and biomarkers were gathered at multiple time point. For animals in the chronic study an additional time point was collected at 28 days. At the end of the study period, the baboons were again anesthetized for measurements and thereafter sacrificed with an overdose of pentobarbital. This study was approved by the Institutional Animal Care and Use Committee at Biocon Research Institute and animals were treated according to NIH guidelines.

### Model framework and description

A simplified mechanistic model of IL-8 mediated activation of CXCR-1/2 receptors and neutrophil response to a pathogen was developed based on available literature information and general knowledge of acute inflammatory response. Receptor level dynamics and systemic parameters were coupled with multiple neutrophil phenotypes to generate dynamic populations of activated neutrophils which reduce pathogen load, and/or primed neutrophils which cause adverse tissue damage when misdirected. Mathematical representation of the interactions detailed in Fig 1 were generated using ordinary differential equation (ODE) framework with the rate of interactions described by mass action kinetics or Hill type kinetics [53,54]. The interactions included in the model gives rise to 16 ODE state variables and 43 rate parameters.

In brief, the model is initiated by a pathogen load, which represents a bacterial inoculation. Presence of pathogen leads to continued growth as well as IL-8 and fMLP cytokine production. IL-8 is generated indirectly from pathogen generation from responding phagocytic mononuclear cells [55]. IL-8 initiates CXCR-1/2 activation in the receptor level, which in turn generates neutrophil phenotype change. Depending on phenotype, neutrophils may cause either pathogen elimination or misdirected tissue damage. A systemic damage indicator represents overall patient health. Increased systemic damage results in further IL-8 generation [56,57], resulting in a positive feedback loop. This simplified system captures the basic functionality of acute IL-8 mediated immune response to pathogen and is capable providing valuable feedback on potential therapeutic treatments modulating these mechanisms. A more detailed description of model equations follows.

#### Pathogen

Eq (1) describes the population of foreign pathogen. Base pathogen growth rate increases linearly with pathogen until approaching a carrying capacity at elevated pathogen loads. In addition to basal pathogen death, the Neutrophil kill/migrate phenotype is capable of decreasing pathogen population through diapedesis, followed by targeted phagocytosis [11,58,59].

$$\frac{dP}{dt}=k_{PG}P-k_{P-N_{K/M}}\;N_{K/M}P-\frac{k_{p}P}{k_{p}^{d}+P}-k_{pL}P^{2}$$ (1)

#### Ligands: Interleukin-8 (IL-8) and fMLP

In the model, neutrophils progress through multiple phenotypes which dictate neutrophil migratory, phagocytic, and antibiotic activity. The association of chemokine IL-8 with the surface CXCR-1/2 triggers the transition of basal neutrophils to functional phenotypes. Previously characterized receptor surface activation, internalization, and recycling rates of CXCR-1/2 are utilized to predict receptor levels and neutrophil phenotypes in response to systemic IL-8 stimulation [14,60]. IL-8 production rate is a function of elevated pathogen and tissue damage [61,62]. Both terms are represented as Hill Equations in Eq (2). While IL-8 is not directly linked to pathogen levels, this simplified representation captures IL-8 release from macrophages and endothelial cells in response to infection.

$$\frac{dC_{IL8}}{dt}=\frac{k_{IL8-P}P}{k_{IL8-P}^{d}+P}+\frac{k_{IL8-D}D^{2}}{k_{IL8-D}^{d}+D^{2}}-k_{IL8}C_{IL8}$$ (2)

Eq (3) characterizes a general pro-inflammatory pathway, which is independent of CXCR-1/2 activation has been added to represent alternate means of neutrophil induced pathogen activation. This generic pathway is not modeled using receptor level dynamics and directly transitions the N_Basal_ (N_B_) population to N_Killing/Migratory_ (N_K/M_).The generic proinflammatory ligand growth is dictated by pathogen level.

$$\frac{dC_{fMLP}}{dt}=\frac{k_{fMLP}P}{k_{fMLP}^{d}+P}-k_{fMLP-D}C_{fMLP}$$ (3)

#### Neutrophil Surface Receptors CXCR-1 & CXCR-2

Receptor level dynamics dictate neutrophils advancement into one of four phenotypes depending on CXCR-1/2 surface activation. Each receptor can occupy one of the three states, namely free surface receptor, surface receptor bound to IL-8 and internalized receptor bound to IL-8 [63]. Eq (4) and Eq (5) describe CXCR-1 surface and internalized populations, which have been non-dimensionalized to remove the free receptor state. Equivalent equations are present for CXCR-2. The active surface state was modeled as the dynamic condition which drives neutrophil population phenotype change [64]. This model makes the assumption that CXCR-1/2 receptors are conserved.

$$\frac{{\text{dC}}_{\text{R}1\text{s}}}{\text{dt}}={\text{k}}_{\text{f}1}{\text{C}}_{\text{IL}8}\left(1-{\text{C}}_{\text{R}1\text{s}}-{\text{C}}_{\text{R}1\text{i}}\right)-{\text{k}}_{\text{r}1}{\text{C}}_{\text{R}1\text{s}}-{\text{k}}_{\text{i}1}{\text{C}}_{\text{R}1\text{s}}$$ (4)

$$\frac{dC_{R1i}}{dt}=k_{i1}C_{R1s}-k_{i1'}C_{R1i}$$ (5)

#### Neutrophil Phenotype

Eq (6) represents the resting state (N_B_) represents basal neutrophils which have not been stimulated by IL-8 or other proinflammatory stimuli. These neutrophils are mobile in blood, but not capable of causing systemic damage or utilizing their anti-pathogen capacity without transitioning to another phenotype. All neutrophils begin in this basal state prior to activation and priming. Without stimulation, neutrophil growth and death rates are in equilibrium, however growth rate increases with the introduction of pathogen, which has been expressed through a filter equation to produce a physiologic time delay [11,65]. CXCR-1/2 surface complex levels dictate the transition rates of N_B_ to the N_Migratory_ (N_M_) or N_killing_ (N_K_) phenotypes. Additionally, there is a direct pathway to transition N_B_ to N_K/M_. This mechanism represents a general proinflammatory process independent of CXCR-1/2 signaling. A filter equation was generated in Eq (7). This function fits the physiologic delay between pathogen generation and increased neutrophils entering circulation.

$$\begin{array}{c} \frac{dN_{B}}{dt}=k_{NG}\left(1+\frac{k_{N_{B}-G}F}{k_{N_{B}-G}^{d}+F}\right)-k_{N_{K-IL8}}N_{B}C_{R1s}\\ \;-k_{N_{M-IL8}}N_{B}C_{R2s}-k_{N_{B}}N_{B}-k_{fMLP-N_{B}}N_{B} \end{array}$$ (6)

$$\frac{dF}{dt}=k_{filter\_on}P-k_{filter\_off}F$$ (7)

Eq (8) contains neutrophils which have been activated via IL-8 mediated CXCR-2 stimulation.

$$\frac{dN_{M}}{dt}=k_{N_{M}-IL8}N_{B}C_{R2s}-k_{N_{M}-N_{K}-IL8}N_{M}C_{R1s}-k_{N_{M}}N_{M}$$ (8)

Eq (9) characterizes the killing phenotype (N_K_), representing neutrophils which have been activated via IL-8 mediated CXCR-1 stimulation. N_K_ neutrophils are capable of untargeted cytotoxic activity, resulting in systemic organ damage. The CXCR-1/2 surface population dictates transition rates into phenotypes. Neutrophil elastase / α1-PI complex was utilized in the model to fit N_K_ neutrophil population. As shown in Eq (10) levels of neutrophil elastase / α1-PI complex equate to levels of circulating N_K_ phenotypes.

$$\frac{dN_{K}}{dt}=k_{N_{K}-IL8}N_{B}C_{R1s}-k_{N_{K}-N_{M}-IL8}N_{K}C_{R2s}-k_{N_{K}}N_{K}$$ (9)

$$\frac{dC_{elas}}{dt}=k_{NE}\frac{dN_{K}}{dt}$$ (10)

Both N_M_ and N_K_ phenotypes are capable of progressing to the N_K_/_M_ phenotype through CXCR-1/2 surface receptor activation. This neutrophil state (N_K/M_), shown in Eq (11), represents neutrophils which have been activated through both CXCR-1 and CXCR-2 and are capable of target pathogen removal, effectively fighting infection. The pathogen equation (Eq (1)) contains a term which dictates pathogen death in response to N_K/M_ levels. Once activated through CXCR-1/2 neutrophils are not capable of returning to the basal N_B_ phenotype.

$$\frac{dN_{K/M}}{dt}=k_{N_{K}-N_{M}-IL8}N_{K}C_{R2s}+k_{N_{M}-N_{K}-IL8}N_{M}C_{R1s}-k_{N_{K/M}}N_{K/M}+k_{fMLP-N_{B}}N_{B}$$ (11)

#### Damage

A systemic damage indicator (Eq (12)) was developed to represent overall animal health. Damage is increased by the population of N_K_ and decays gradually as tissue and organs recover. Creatinine, a biomarker for kidney function, was utilized in Eq (13) as an indicator for the damage term ensemble computation. Creatinine is maintained at a constant level in the absence of damage, but systemic levels increase with damage as body’s ability to clear creatinine decreases [66].

$$\frac{dD}{dt}=k_{D-N_{K}}N_{K}\left(1-D\right)-k_{D}D$$ (12)

$$\frac{dC_{creat}}{dt}=k_{creat-P}-k_{creat}\left(1-D\right)C_{creat}$$ (13)

The model uses empirical time series of Pathogen (CFU), IL-8 (nM), creatinine (mM), White Blood Cell count (10^3^ cells/μl), and neutrophil elastase / α1-PI complex (ng/ml) for computation of the ensemble. State variables and their initial conditions are listed in Table 1.

### Parameter estimation

The model contains 38 parameters, 13 of which are fixed based on literature data (Table 1). Parameter values were inferred using a Bayesian parallel tempering approach [22,67], which utilizes traditional Markov Chain Monte Carlo (MCMC) methods to sample the Bayesian posterior distribution P(**p**|**y**), the probability of parameter set **p** given data **y**, given by the Bayes formula

$$P\left(p|\;y\right)=\frac{L(y|p)\theta \left(p\right)}{\int L(Y|\;p)\theta \left(p\right)}$$

where L(**y**|**p**) is the likelihood of observing **y** for a model with parameters **p**, θ(**p**) is the prior distribution, and ∫*L*(*Y* | ***p***)*θ*(***p***) is the normalizing constant. Additional sampling efficiency is gained by running multiple parallel chains evolving at different temperatures. Higher temperature increases the likelihood of acceptance of proposed steps. This allows the high temperature chains to move more freely through the parameter space, avoiding getting stuck in local minima. This results in more efficient exploration of parameter space [20,68] a method we have applied extensively in parameter estimation of practically unidentifiable complex non-linear models [10,69,70]. This resulted in the creation of parameter ensembles, where each parameter is represented by a posterior distribution, rather than a single value. Free parameters were fit separately to the survivor and non-survivor experimental data sets, resulting in two parameter ensembles representing surviving and non-surviving animals.

#### Bayesian priors

Prior distributions were selected for each parameter. In each case uniform priors were used, with a suitably large range so as to encompass all reasonable parameter values. This was ideal due to the limited prior knowledge and phenomenological nature of many of the parameters. Tighter ranges were enforced on select parameters as required to avoid non-physiologic model behavior. All candidate parameter values were selected from these pre-defined priors.

#### Parameter set fitness

Fitness (log likelihood) of candidate parameters sets was determined by the difference between model simulations and experimental data, as determined by the sum of squared residuals cost function,

$$Fitness=\sum_{i,j,k}w_{i,j,k}\;*\frac{{\left(y_{i,j,k}-{\hat{y}}_{i,j,k}\right)}^{2}}{2\sigma_{i,j,k}{}^{2}}$$

Where w_i,j,k_ is a weighting function, y_i,j,k_ is the output for a simulation with a single set of parameters, ${\hat{\text{y}}}_{\text{i},\text{j},\text{k}}$ is the experimental mean, and σ_i,j,k_ σ_i,j,k_ is the experimental standard deviation at time point *i*, observable *j*, and data set *k*. No additional penalties or constraints were added to parameter selection. To ensure proper fitting of the pathogen observable a threshold was added to change all values below the experimental limit of detection (4.4 CFU) to 0.

#### Parallel tempering

To efficiently sample the posterior distribution, six separate Markov chains were run, initiated with parameter values randomly selected from the supplied prior distributions which met a maximum energy criterion. Each chain was initiated with a temperature and step size parameter which controlled the chain’s ability to fully explore the parameters space. Chains were allowed to swap from a higher temperature to a lower temperature every 25 steps to allow for local sampling of newly found local minima. Step size and temperature parameters dynamically changed every 6,250 and 2,500 steps respectively to attempt to reach an ideal step acceptance rate of 23% [71], and swap rates of 15%-30%. Once these targets were reached, the temperature schedule and step sizes were fixed. Parameter sets were saved every 25 steps. Full exploration of parameter space was confirmed by examining, for each parameter, the frequency histogram of its full marginal posterior distribution, confirming that it spanned the prior domain.

We measured convergence and chain stationarity using the Gelman-Rubin criteria [72,73]. All parameters had converged with a potential scale reduction factor (PSRF) < 1.1 following 200,000 (x25) MCMC steps. Another 100,000 steps were taken to build a posterior distribution for each parameter that would be used for all model analysis and simulation. This ensured that all samples from the burn-in time for each chain were discarded, and only samples from the correct stationary distribution were used. The ensemble of all parameter sets from the lowest chain comprised the computed ensemble (posterior distribution).

### Selection of key parameters

In order to better capture the underlying biological differences between animals that survive and those that die following the same challenge we attempted to identify the most important parameters in determining animal fate. After computing ensembles for survivors and non-survivors, we performed regularized logistic regression, forward conditional stepwise logistic regression, and backward conditional logistic regression to identify a subset of parameters that are most indicative of outcome. Predictors consisted of all estimated parameters of both ensembles, and the indicator variable was the source (survivor or non-survivor) of the ensemble. Parameters were selected that were considered significant by all three methods, leaving a set of seven key parameters.

#### Model fitting

A second round of model fitting was then performed. In this round, 18 of the 25 parameters were fit simultaneously to both data sets, resulting in identical parameter values in the two ensembles. The seven parameters identified as being significant were fit twice, once against the survivor data set and once against the non-survivor data set, resulting in different parameter values across the two ensembles. This resulted in a smaller and more focused difference between the final ensembles.

### Global sensitivity analysis

Global Sensitivity analysis was done to determine the independent and correlated contributions of rate parameters on cumulative damage. Area under the damage curve was chosen as the system output. To reduce the computational cost of GSA, Random Sampling High Dimensional Model Representation (RS-HDMR) approach was used [74]. Here, a multivariate output function (eg. AUC_D_) was approximately represented by weighted optimal expansion functions (called as component functions). The expansion coefficients of these functions were determined by least-squares regression simultaneously from one set of Monte Carlo samples. In general, for input vector, $\bar{x}=[x_{1},\;x_{2},\;\ldots ,\;x_{n}]$ of rate parameters, in an *n*–dimensional space, a multivariate output function, $f(\bar{x})$, is approximated by a sum of terms including the mean (*f*_0_) and the component functions (*g*_*l*_). Mathematically,

$$f(\bar{x})=f_{0}+\sum_{l=1}^{2^{n}-1}g_{l}$$

Here, the index *l* indicates all possible combinations of the input parameters. In practice, not all component functions are significant and an F-test can be used to determine which component function should be excluded from the expansion [75]. For our work, we evaluated the variance based Sobol’ indices using these component functions. The workflow adopted here starts with generation of Monte Carlo samples of the rate parameters from the ensembles obtained by the parallel tempering approach. Since they come from the ensemble, information on the covariance between the parameter distributions for the population of survivors and non-survivors is retained. Next, a detailed procedure is followed which includes simultaneous construction of all the component functions, removal of non-significant component functions using an F-test ratio score, re-evaluation of component functions and finally evaluation of the Sobol’ sensitivity indices. The first order Sobol’ sensitivity indices which capture the influence of a single parameter (but averaged over the other parameters) are defined as:

$$S_{l}=\frac{Cov(f(\bar{x}),g_{l}(\bar{x}))}{\sigma^{2}},\;l=1,\;2,\;3,\ldots .,\;n$$

Here, *σ* ^2^ is the total variance in the output and *Cov*(•) is the covariance between the output function and each of the first order component functions. For clarity, the component function, *g*_*l*_, is written as a function of $\bar{x}$ but in reality it is only a function of the input parameter for which it is defined (for example, *x*_*l*_) and not the entire vector. Further, this sensitivity index is a sum of two terms that capture independent ($S_{l}^{a}$) and correlated contributions ($S_{l}^{b}$) of the input, which are defined as:

$$S_{l}^{a}=\frac{\langle g_{l}(\bar{x}),g_{l}(\bar{x})\rangle }{\sigma^{2}}$$

and

$$S_{l}^{b}=\frac{\sum_{\begin{array}{l} k=1\\ k\neq l \end{array}}^{n}\langle g_{l}(\bar{x}),g_{k}(\bar{x})\rangle }{\sigma^{2}}.$$

The inner products, 〈•〉, are defined as:

$\langle g_{k}(\bar{x}),g_{l}(\bar{x})\rangle =\int_{x_{1}}\ldots \int_{x_{n}}w(\bar{x})g_{k}(\bar{x})g_{l}(\bar{x})dx_{1}\ldots \;dx_{n}$ and $w(\bar{x})$ is the probability density function of the inputs informed by the parameter ensembles. Similar equations can be written for the higher order component functions and sensitivity indices. Further details on the evaluation of the component function for various types of models are given in [74,76,77]. To determine the importance of a given parameter, it is necessary to combine all the important sensitivity indices (all orders) into a total sensitivity index, which for a parameter *i* can be defined as:

$$S_{T_{i}}=S_{i}+\sum_{\begin{array}{l} j=1\\ j\neq i \end{array}}^{n}S_{ij}+\sum_{\begin{array}{l} j<k=1\\ j,k\neq i \end{array}}^{n}S_{ijk}+\ldots ...$$

For most systems, very high order interactions are negligible and therefore, indices until the third order are sufficient, with most systems requiring only until the second order terms [74]. In this work, we constructed a third order RS-HDMR. All GSA computations were performed using the ExploreHD software (Aerodyne Research Inc., MA, USA).

### Treatment framework

#### Treatment implementation

After model fitting and analysis, a potential extracorporeal treatment was introduced (See Fig 7). The extracorporeal treatment directly modulates CXCR-1/2 levels of circulating neutrophils, limiting passage of N_B_ to N_K_ and N_M_. This mechanism of limiting CXCR-1/2 surface levels is modeled solely in the receptor level equations of the model. A heaviside function is used to turn treatment on and off at various treatment times. The *k*_*ft*1_ parameter represents treatment effectiveness, which combines device size, efficacy, efficiency and flow rate. Eq (14) is the modified CXCR-1 surface receptor equation which includes the Heaviside function. Eq (15) characterizes the trapped receptor state of CXCR-1. Similarly Eq (16) and Eq (17) are constructed for CXCR-2 and its associated trapped receptor state.

$$\begin{array}{l} \frac{dC_{R1s}}{dt}=k_{f1}C_{IL8}\left(1-C_{R1s}-C_{R1i}-C_{R1t}\right)-k_{r1}C_{R1s}-k_{i1}C_{R1s}+k_{ft1'}C_{R1t}\\ \;-Heaviside\left(t,k_{treat-on},k_{treat-off}\right)k_{ft1}\left(1-C_{R1s}-C_{R1i}-C_{R1t}\right) \end{array}$$ (14)

$$\frac{{\text{dC}}_{\text{R}1\text{t}}}{\text{dt}}=\text{Heaviside}\left(\text{t},{\text{k}}_{\text{treat}-\text{on}},{\text{k}}_{\text{treat}-\text{off}}\right){\text{k}}_{\text{ft}1}\left(1-{\text{C}}_{\text{R}1\text{s}}-{\text{C}}_{\text{R}1\text{i}}-{\text{C}}_{\text{R}1\text{t}}\right)-{\text{k}}_{\text{ft}1'}{\text{C}}_{\text{R}1\text{t}}$$ (15)

$$\begin{array}{l} \frac{dC_{R2s}}{dt}=k_{f2}C_{IL8}\left(1-C_{R2s}-C_{R2i}-C_{R2t}\right)-k_{r2}C_{R2s}-k_{i2}C_{R2s}+k_{ft2'}C_{R2t}\\ \;-Heaviside\left(t,k_{treat-on},k_{treat-off}\right)k_{ft2}\left(1-C_{R2s}-C_{R2i}-C_{R2t}\right) \end{array}$$ (16)

$$\frac{{\text{dC}}_{\text{R}2\text{t}}}{\text{dt}}=\text{Heaviside}\left(\text{t},{\text{k}}_{\text{treat}-\text{on}},{\text{k}}_{\text{treat}-\text{off}}\right){\text{k}}_{\text{ft}2}\left(1-{\text{C}}_{\text{R}2\text{s}}-{\text{C}}_{\text{R}2\text{i}}-{\text{C}}_{\text{R}2\text{t}}\right)-{\text{k}}_{\text{ft}2'}{\text{C}}_{\text{R}2\text{t}}$$ (17)

#### Classification of patient outcome

In order to implement and evaluate treatment frameworks, simulated patient survivorship needed to be explicitly labeled. This was accomplished using a logistic regression classifier as specified by the machine learning software Weka [78]. The estimated parameter ensemble was partitioned into a training set and test set to build the classifier, using 20% of the ensemble as training data. Two features were used for training, total accumulated damage measured by area under the curve of the damage time course for each patient, as well as the peak damage experienced by the patient. Training with these features resulted in a classifier that could label a patient as surviving or dying after being exposed to a specific infection and possible treatment.

## Supporting Information

S1 Fig Model predictions of receptor dynamics following infection. Mean (red), 25^th^-75^th^ percentile (dark blue), and 5^th^-95^th^ percentile trajectories of the simulated ensemble are shown. Predictions are shown for the CXCR1 **(A-B)** and CXCR2 **(C-D)** bound to IL-8 and actively signaling from the cell surface, as well as internalized and unable to signal.
(TIF)

S2 Fig Effects of varying Nk decay rates on simulated treatment. Survival rates of a simulated population of animals following treatment with the proposed extracorporeal device considering a device-receptor affinity of 1x10-3 M for k_Nk values of **(A)** 50% above, **(B)** 10% above, **(D)** 10% below, and **(E)** 50% below the baseline vale **(C)**. In all cases the time of treatment was varied between 0 and 10 hours post infection and ended between 0 and 100 hours post infection.
(TIF)

S3 Fig Effects of varying Nk induction rates on simulated treatment. Survival rates of a simulated population of animals following treatment with the proposed extracorporeal device considering a device-receptor affinity of 1x10-3 M for k_Nk_IL8 values of **(A)** 50% above, **(B)** 10% above, **(D)** 10% below, and **(E)** 50% below the baseline estimated value **(C)**. In all cases the time of treatment was varied between 0 and 10 hours post infection and ended between 0 and 100 hours post infection.
(TIF)

S1 Dataset Experimental data. Sixteen baboons (*Papio ursinus*) weighing between 19 and 32 kg were infused with 2 x 109 CFU *Escherichia coli* per kg over a two-hour period and followed until predetermined time points or death.
(XLS)
