# Supplementary figures and images for: A Neutrophil Phenotype Model for Extracorporeal Treatment of Sepsis
Source: PLoS Comput Biol. 2015 Oct 15;11(10):e1004314. doi: 10.1371/journal.pcbi.1004314 (PMC4607502; doi:10.1371/journal.pcbi.1004314)

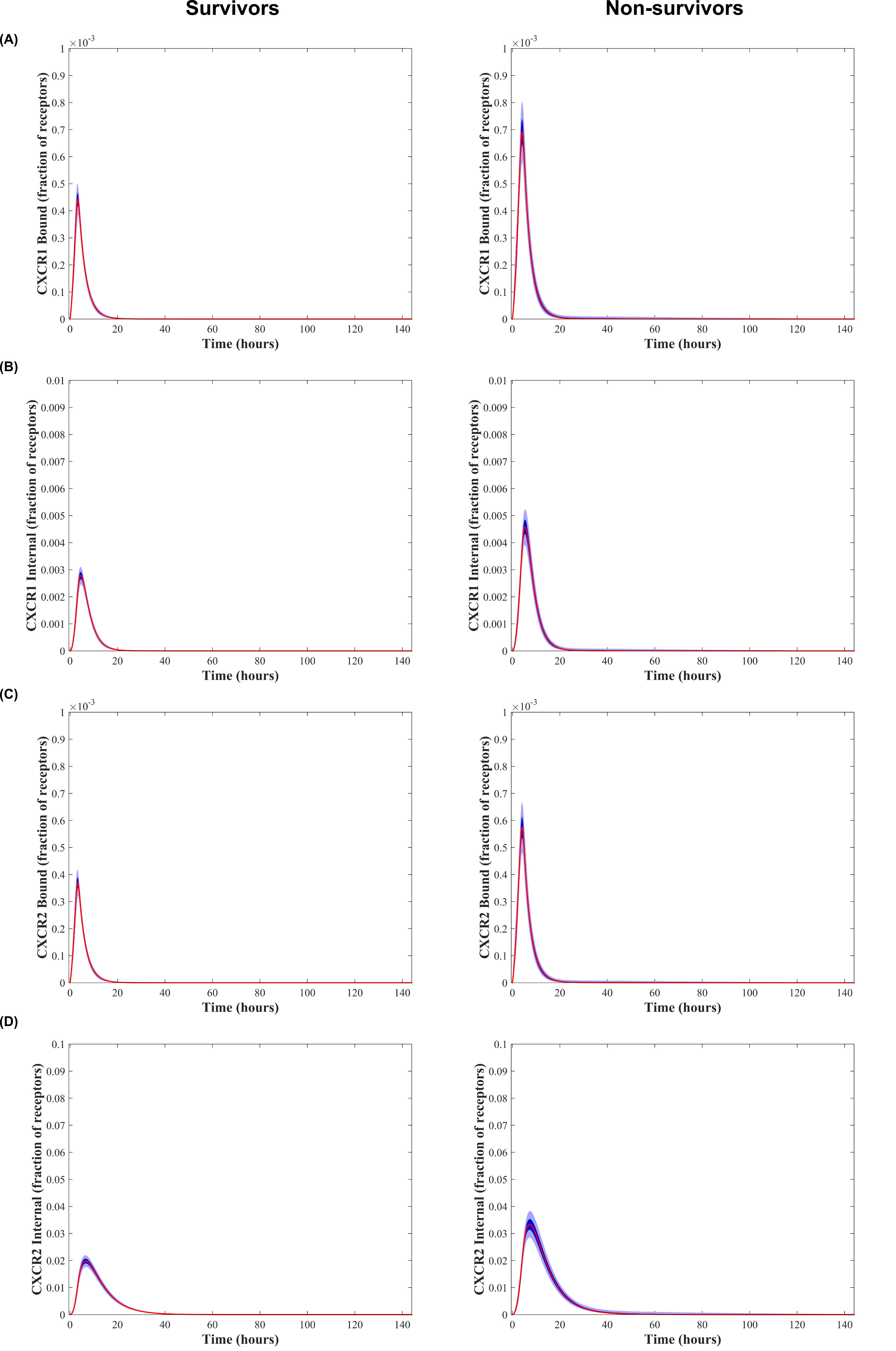

Supplement: S1 Fig — Mean (red), 25th-75th percentile (dark blue), and 5th-95th percentile trajectories of the simulated ensemble are shown. Predictions are shown for the CXCR1 (A-B) and CXCR2 (C-D) bound to IL-8 and actively signaling from the cell surface, as well as internalized and unable to signal. (TIF) [file pcbi.1004314.s001.tif]

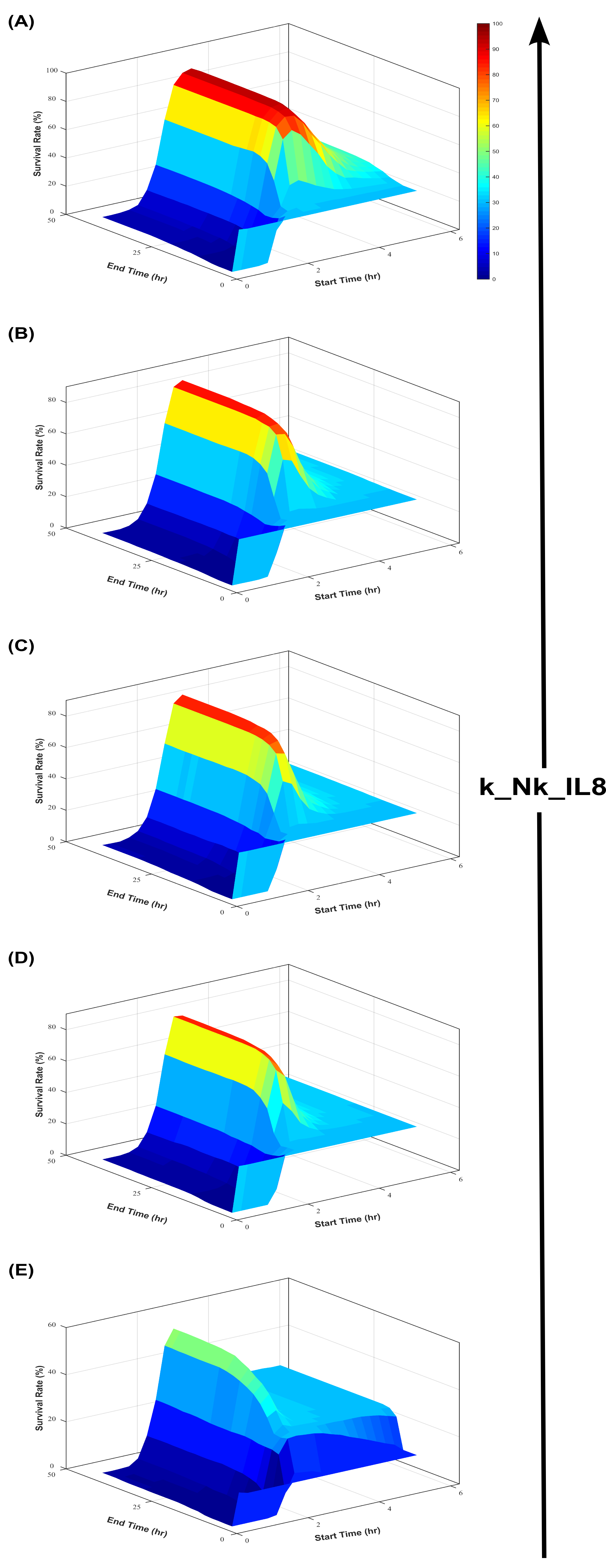

Supplement: S2 Fig — Survival rates of a simulated population of animals following treatment with the proposed extracorporeal device considering a device-receptor affinity of 1x10^-3^ M for k_Nk values of (A) 50% above, (B) 10% above, (D) 10% below, and (E) 50% below the baseline vale (C). In all cases the time of treatment was varied between 0 and 10 hours post infection and ended between 0 and 100 hours post infection. (TIF) [file pcbi.1004314.s002.tif]

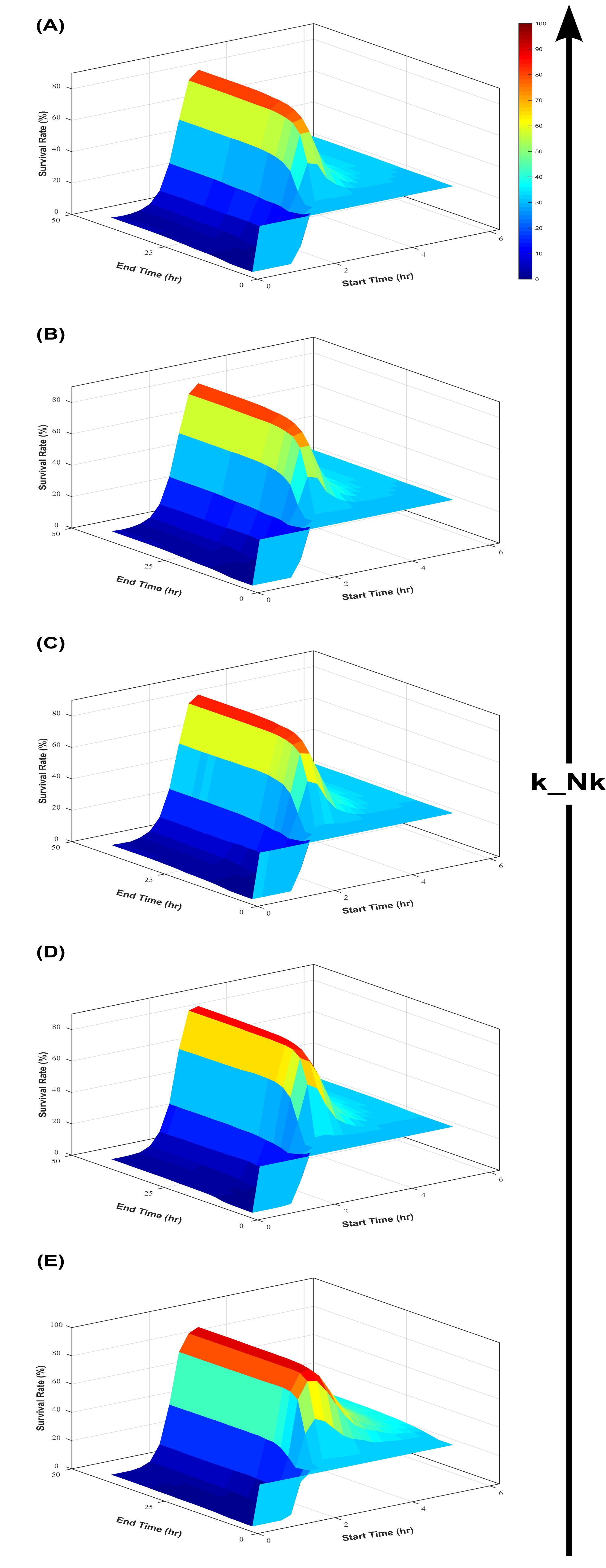

Supplement: S3 Fig — Survival rates of a simulated population of animals following treatment with the proposed extracorporeal device considering a device-receptor affinity of 1x10^-3^ M for k_Nk_IL8 values of (A) 50% above, (B) 10% above, (D) 10% below, and (E) 50% below the baseline estimated value (C). In all cases the time of treatment was varied between 0 and 10 hours post infection and ended between 0 and 100 hours post infection. (TIF) [file pcbi.1004314.s003.tif]
